# Internal migration of physicians who graduated in Brazil between 1980 and 2014

**DOI:** 10.1186/s12960-018-0286-8

**Published:** 2018-05-02

**Authors:** Mario Cesar Scheffer, Alex Jones Flores Cassenote, Aline Gil Alves Guilloux, Mario Roberto Dal Poz

**Affiliations:** 1 Preventive Medicine Department, Medical School of University of São Paulo, São Paulo, Brazil; 2 Institute of Social Medicine, University of Rio de Janeiro State, Rio de Janeiro, Brazil

**Keywords:** Human resources for health, Distribution of physicians, Medical schools, Internal migration, Brazil

## Abstract

**Background:**

The internal migration of physicians from one place to another in the same country can unbalance the supply and distribution of these professionals in national health systems. In addition to economic, social and demographic issues, there are individual and professional factors associated with a physician’s decision to migrate. In Brazil, there is an ongoing debate as to whether opening medicine programmes in the interior of the country can induce physicians to stay in these locations. This article examines the migration of physicians in Brazil based on the location of the medical schools from which they graduated.

**Methods:**

A cross-sectional design based on secondary data of 275,801 physicians registered in the Regional Councils of Medicine (Conselhos Regionais de Medicina—CRMs) who graduated between 1980 and 2014. The evaluated outcome was migration, which was defined as moving away from the state where they completed the medicine programme to another state where they currently work or live.

**Results:**

57.3% of the physicians in the study migrated. The probability of migration ratio was greater in small grouped municipalities and lower in state capitals. 93.4% of the physicians who trained in schools located in cities with less than 100,000 inhabitants migrated. Fewer women (54.2%) migrated than men (60.0%). More than half of the physicians who graduated between 1980 and 2014 are in federative units different from the unit in which they graduated. Individual factors, such as age, gender, time of graduation and specialty, vary between the physicians who did or did not migrate.

**Conclusions:**

The probability of migration ratio was greater in small municipalities of the Southeast region and strong in the states of Tocantins, Acre and Santa Catarina. New studies are recommended to deepen understanding of the factors related to the internal migration and non-migration of physicians to improve human resource for health policies.

**Electronic supplementary material:**

The online version of this article (10.1186/s12960-018-0286-8) contains supplementary material, which is available to authorized users.

## Background

The internal migration of physicians who leave a city or region of the country to establish themselves in another city or region of the same country can unbalance the distribution of these professionals in the national territories [1]. Relatively few studies on the various forms of internal migration of physicians and other health professionals are available in the international literature.

Studies on the mobility of physicians between countries identified a convergence of individual and professional motivations as well as policy, economic, social and demographic issues [2–4]. Similar to the determinants of the mobility of skilled workers in general [5], physicians most commonly migrate from places with worse living and working conditions and with few opportunities for professional development. The attractiveness of specialized education centres, career prospects [6, 7], better pay [8, 9] and age [10] plays a relevant role in the decision of physicians to migrate. For instance, young physicians are more inclined to change cities or countries.

In Brazil, recent policies have focused on the number, supply, demand, distribution and training of physicians. Since 2013 with the institution of the More Doctors law [11], new training programmes have opened with expanded graduate vacancies in medicine, expanded medical residency programs and the admission of foreign and Brazilian physicians to practice in underserved municipalities and regions.

The “interiorization of medical training” is the decentralization of medical education resources to cities and regions where there are no or few medical schools are considered a possible inducing factor for physicians to stay in these localities [12]. There are no studies in Brazil that support this relationship, although physicians tend to remain in the cities where they conclude their medical residency [13] and these programmes are more concentrated in the South and Southeast regions of Brazil [14].

The objective of the present study is to evaluate the profile and distribution of physicians trained in Brazil between 1980 and 2014. We considered the federative units and the cities where the professionals graduated and where they currently work or reside to contribute to the understanding of the internal migration of physicians in the country.

## Methods

This was cross-sectional study using secondary data from the Medical Demographics in Brazil [15]. This survey is the census that includes administrative records from: Regional Medicine Councils (Conselhos Regionais de Medicina—CRMs); National Residency Commission (Comissão Nacional de Residência—CNRM) of the Brazilian Ministry of Education (Ministério da Educação—MEC); and the Brazilian Medical Association (Associação Médica Brasileira—AMB).

The CRMs are responsible for registration and inspection of physicians and their professional activity. All the active physicians in Brazil must be registered in a medical council; there are 27 medical councils, one in each Brazilian Federal Unit, which together integrated the Federal Medicine Council’s (Conselho Federal de Medicina—CFM) database.

All physicians registered in the CRMs who completed a degree in medicine between 1980 and 2014 were included in the study. The analysed time interval, which encompassed a significant contingent of graduated and enrolled physicians, included public or private degree programmes in various locations.

The initial database was composed of 388,203 physicians, of which 20% (77,569) did not match the temporal inclusion criteria. Of the 310,634 physicians considered, those with absent or incomplete information concerning current address or graduating school place were excluded, which led to the exclusion of 12.2% of the physicians (34,828). The final database included 275,801 physicians that make up the universe of physicians who graduated in Brazil between 1980 and 2014 (Table 1). The physicians’ places of graduation (medical school headquarter city), the dates of the first records of the physicians in the CRM (primary enrolment) and the current addresses provided by the physicians to the CRM, which corresponded to a residence and/or work address, were considered.

**Table 1:** Physicians who graduated in Brazil between 1980 and 2014 according to the location of the graduating school in the federative units

Federative units	Number	Percent
São Paulo	55 874	20.26
Rio De Janeiro	50 480	18.30
Minas Gerais	39 065	14.16
Rio Grande do Sul	24 603	8.92
Parana	13 987	5.07
Bahia	11 528	4.18
Pernambuco	9 934	3.60
Pará	7 704	2.79
Espírito Santo	7 550	2.74
Ceará	7 238	2.62
Santa Catarina	6 959	2.52
Paraiba	6 237	2.26
Alagoas	4 359	1.58
Goiás	3 850	1.40
Amazonas	3 521	1.28
Rio Grande Do Norte	3 317	1.20
Federal District	3 151	1.14
Maranhão	3 085	1.12
Piauí	2 883	1.05
Mato Grosso Do Sul	2 737	0.99
Sergipe	2 291	0.83
Mato Grosso	2 177	0.79
Tocantins	1 804	0.65
Rondônia	908	0.33
Roraima	318	0.12
Acre	241	0.09
Amapá	0	0.0
Total	275 801	100.00

The evaluated primary outcome of interest was migration. The binary variable (did not migrate/migrated) was defined as a move from one state (state of the school where the physician completed the medical degree) to another state (current living or working location). The qualitative nominal and ordinal variables were evaluated as factors and referred to the characteristics of the physicians (age, gender, date of graduation, and specialty) and the medical programmes (geographical location and public or private institution).

The variable “type of municipality of the medical school” was stratified into three levels based on the 2010 census conducted by the Brazilian Institute of Geography and Statistics [15, 16] according to the population strata as follows: (1) capitals, (2) large interior municipalities (LM interior) with 100,000 or more inhabitants and (3) small interior municipalities (SM interior) with less than 100,000 inhabitants.

The migration according to the type of municipality of the medical school was analysed using a probability migration ratio (PMR) like indicator obtained from the ratio between the probability of the physician migrating following graduation from a school located in the capitals (reference category) divided by the probability of the physician migrating following graduation from a school located in the interior (LM interior and SM interior).

The Statistical Package for the Social Sciences (SPSS) 20 for Windows (International Business Machines Corp., Armonk, NY, USA) was used for the analysis. The map was generated with the aid of QGIS Geographic Information System version 2.4.0-Chugiak (QGIS Development Team 2016. Open Source Geospatial Foundation Project: http://qgis.org/en/site/).

This study was approved by the Ethics in Research Committee of the Medical School of the University of São Paulo (Universidade de São Paulo) (Opinion n. 797,424, 09/03/2014).

## Results

**Fig. 1:**
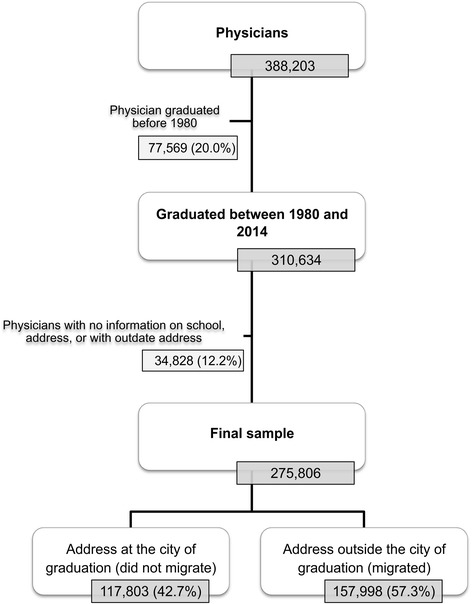
Flowchart of the study population

Among the 275,801 total physicians considered, 157,998 (57.3%) migrated (i.e., were living and/or working in a different state than the state where the medical school from which they graduated was located at the time of the study) (Table 1 and Fig. 1).

From the total physicians analysed, 52.9% were male, 22.8% were below 35 years of age, 22.5% had graduated at least 5 years ago and 60% had completed their specialization or medical residence in one of the 53 recognized medical specialties. Most of the physicians graduated from public schools (57.9%) located in the Southeast region (55.5%) and the state capitals (56.1%).

From 1980 to 2014, more than half of the Brazilian physicians (52.7%) graduated from schools located in the following three states: São Paulo (55,874 physicians or 20.2% of the total), Rio de Janeiro (50,480 or 18.3%) and Minas Gerais (39,065 or 14.1%). The states with the fewest graduated physicians were Acre (241 or 0.09%), Roraima (318 or 0.12%) and Rondônia (908 or 0.33%). The state of Amapá did not have any graduated physicians registered because it did not have a medical programme during the analysed period (Table 1).

A total of 60.7% of the physicians older than 35 to 40 years of age migrated. In the age range below or equal to 30 years, 52.0% migrated (Table 2). The women migrated less than the men (54.2 and 60.0%, respectively). A greater proportion of physicians who graduated 10 to 15 years before the study migrated (60.1% migrated) compared to the other groups stratified by the time since graduation. Additionally, more physicians without a specialization migrated (59.7%) compared with the specialists (55.7%) and physicians from private medical schools (67.8%) compared with the public medical schools (49.7).

**Table 2:** Physicians who graduated between 1980 and 2014 who migrated and did not migrate according to gender, time since graduation, specialization and characteristics of the graduating schools

	Did not migrate	Percent	Migrated	Percent	Total
Physicians’ characteristics					
Age					
≤ 30 years	25 096	48.0	27 201	52.0	52 297
30–35 years	22 303	41.2	31 805	58.8	54 108
35–40 years	16 521	39.3	25 490	60.7	42 011
40–45 years	13 751	42.2	18 870	57.8	32 621
45–50 years	13 098	42.9	17 419	57.1	30 517
50–55 years	13 497	43.6	17 439	56.4	30 936
55–60 years	10 838	41.7	15 162	58.3	26 000
60–65 years	2 014	36.1	3 572	63.9	5 586
65–70 years	407	37.8	669	62.2	1 076
≥ 70 years	273	42.5	369	57.5	642
Gender					
Female	59 529	45.8	70 421	54.2	129 950
Male	58 274	40.0	87 577	60.0	145 851
Time since graduation					
≤ 5 years	29 591	47.8	32 327	52.2	61 918
5–10 years	19 601	39.2	30 392	60.8	49 993
10–15 years	14 895	39.0	23 264	61.0	38 159
15–20 years	13 627	42.3	18 558	57.7	32 185
20–25 years	13 265	43.2	17 425	56.8	30 690
25–30 years	13 428	43.4	17 523	56.6	30 951
30–35 years	11 990	41.0	17 258	59.0	29 248
≥ 35 years	1 405	52.9	1 248	47.1	2 653
Specialist					
No	43 531	40.3	64 469	59.7	108 000
Yes	74 272	44.3	93 529	55.7	167 801
Total	117 803	42.7	157 998	57.3	275 801
School characteristics					
Greater region					
North	6 903	47.6	7 593	52.4	14 496
Northeast	30 676	60.3	20 196	39.7	50 872
Southeast	54 591	35.7	98 378	64.3	152 969
South	18 704	41.1	26 845	58.9	45 549
Central-West	6 929	58.2	4 986	41.8	11 915
Municipality					
Capital	93 034	60.2	61 624	39.8	154 658
LM interior^a^	23 822	22.3	82 939	77.7	106 761
SM interior^b^	947	6.6	13 435	93.4	14 382
Legal nature of school					
Public	80 356	50.3	79 339	49.7	159 695
Private	37 438	32.2	78 653	67.8	116 091
No information	9	60.0	6	40.0	15
Total	117 803	42.7	157 998	57.3	275 801

^a^LM interior—interior large municipalities ≥ 100 000 inhabitants;

^b^SM interior—interior small municipalities < 100 000 inhabitants

Migration was also analysed according to the location of the graduate schools based on the municipality size and country region. In all regions, PMR was greater in small municipalities and lower in the capitals. Compared to the capitals, PMR of physicians who graduated from schools located in the group of small municipalities was 9.7-fold higher (Table 3).

**Table 3:** Physicians who graduated in Brazil between 1980 and 2014 who migrated and did not migrate according to the location of the graduating school in the Greater Regions and in municipalities grouped by size

	Did not migrate	Percent	Migrated	Percent	Total	PMR
North						
Capital	6 628	52.4	6 027	47.6	12 655	1.00
LM interior	167	17.5	789	82.5	956	3.00
SM interior	108	12.2	777	87.8	885	4.29
Total	6 903	47.6	7 593	52.4	14 496	–
Northeast						
Capital	29 635	62.9	17 461	37.1	47 096	1.00
LM interior	1 041	27.6	2 735	72.4	3 776	2.28
SM interior	0	0.0	0	0.0	0	–
Total	30 676	60.3	20 196	39.7	50 872	–
Southeast						
Capital	36 723	60.3	24 218	39.7	60 941	1.00
LM interior	17 057	21.6	61 921	78.4	78 978	2.79
SM interior	811	6.2	12 239	93.8	13 050	9.70
Total	54 591	35.7	98 378	64.3	152 969	–
South						
Capital	13 200	58.1	9 505	41.9	22 705	1.00
LM interior	5 476	24.2	17 164	75.8	22 640	2.40
SM interior	28	13.7	176	86.3	204	4.24
Total	18 704	41.1	26 845	58.9	45 549	–
Central-West						
Capital	6 848	60.8	4 413	39.2	11 261	1.00
LM interior	81	19.7	330	80.3	411	3.09
SM interior	0	0.0	243	100.0	243	–
Total	6 929	58.2	4 986	41.8	11 915	–
Total	117 803	42.7	157 998	57.3	275 801	–

LM interior—interior large municipalities ≥100 000 inhabitants; SM interior—interior small municipalities < 100 000 inhabitants

*PMR* probability migration ratio

A total of 93.4% of the physicians who graduated from schools located in municipalities with less than 100,000 inhabitants migrated (Table 2). The Southeast region had the largest absolute and relative contingent of physicians who graduated from small municipalities, and 93.8% of these physicians migrated (Table 3). Regarding the nature of the schools, physicians who graduated from private schools migrated more often (67.8%) than physicians who graduated from public schools (49.7%), although in the physicians analysed the physicians who graduated from private schools represented 42.1% of the total.

**Fig. 2:**
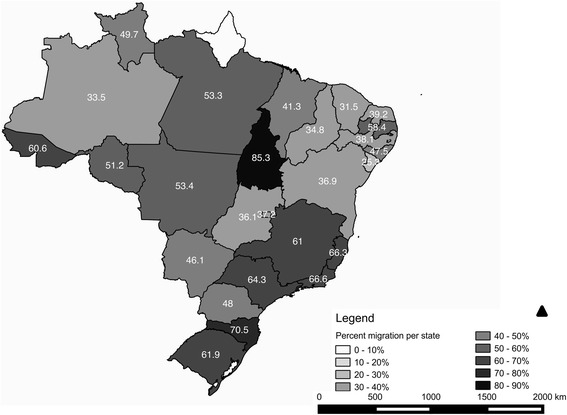
Percentage of trained physicians who migrated to another state per federative unit

Among the Brazilian states, the largest migration ratios were observed in Tocantins (85.3%), Santa Catarina (70.5%), Acre (60.6%) and Rio Grande do Sul (61.9%), whereas Alagoas (25.3%), Ceará (31.5%), Amazonas (33.5%) and Piauí (34.8%) had the smallest migration percentages (Fig. 2).

Table 4 shows the physician population rate (PPR) and the percent of graduated, migrated and immigrated physicians according Brazilian states and populational stratum. In this table, it is possible to evaluate some phenomena: (I) in almost all situations, small municipalities (SM interior) lost more than 85% of medical graduates; (II) from the 16 municipalities (LG interior) that had the potential to train physicians, only 2 of them lost less than 65% of medical graduates; (III) from the 26 capital cities, only 6 lost more than 65% of medical graduates; and (IV) the capital of São Paulo state receives twice as many physicians as it loses.

**Table 4:** Training and migration/immigration of physician in Brazilian municipalities according to states and population strata between 1980 and 2014

State	Stratum	Population	Physician in 2014	PPR^a^	%graduated	%migrated	%immigrated
Rondônia	Capital	484 992	1 283	2.65	0.29%	49.0%	54.6%
	LG interior	229 295	334	1.46	0.00%	0.00%	100.0%
	SM interior	1 013 927	670	0.66	0.04%	68.3%	91.8%
Acre	Capital	357 194	726	2.03	0.09%	60.6%	74.7%
	LG interior	–	–	–	–	–	–
	SM interior	419 269	175	0.42	0.00%	0.00%	100.0%
Amazonas	Capital	1 982 177	4 123	2.08	1.28%	33.5%	20.7%
	LG interior	109 225	31	0.28	0.00%	0.00%	100.0%
	SM interior	1 716 519	212	0.12	0.00%	0.00%	100.0%
Roraima	Capital	308 996	657	2.13	0.12%	49.7%	63.9%
	LG interior	–	–	–	–	–	–
	SM interior	179 076	20	0.11	0.00%	0.00%	100.0%
Para	Capital	1 425 922	5 333	3.74	2.79%	53.3%	5.1%
	LG interior	2 435 197	1 341	0.55	0.00%	0.00%	100.0%
	SM interior	4 138 610	617	0.15	0.00%	0.00%	100.0%
Amapa	Capital	437 256	621	1.42	0.00%	0.00%	100.0%
	LG interior	108 897	29	0.27	0.00%	0.00%	100.0%
	SM interior	188 843	23	0.12	0.00%	0.00%	100.0%
Tocantins	Capital	257 904	840	3.26	0.02%	64.2%	96.1%
	LG interior	164 093	548	3.34	0.35%	82.5%	53.1%
	SM interior	1 056 167	762	0.72	0.28%	90.4%	83.3%
Maranhao	Capital	1 053 922	3 327	3.16	1.09%	40.1%	22.8%
	LG interior	1 185 668	989	0.83	0.03%	90.5%	98.6%
	SM interior	4 554 711	634	0.14	0.00%	0.00%	100.0%
Piaui	Capital	836 475	3 714	4.44	1.05%	34.8%	23.6%
	LG interior	148 832	188	1.26	0.00%	0.00%	100.0%
	SM interior	2 198 859	675	0.31	0.00%	0.00%	100.0%
Ceara	Capital	2 551 806	8 684	3.40	2.33%	25.0%	25.3%
	LG interior	1 491 079	1 470	0.99	0.30%	82.8%	86.3%
	SM interior	4 735 691	1 331	0.28	0.00%	0.00%	100.0%
RG Do Norte	Capital	853 928	3 808	4.46	1.16%	38.1%	26.4%
	LG interior	509 728	747	1.47	0.04%	70.6%	93.9%
	SM interior	2 010 303	472	0.23	0.00%	0.00%	100.0%
Paraiba	Capital	769 607	4 291	5.58	1.55%	54.6%	19.7%
	LG interior	637 589	1 678	2.63	0.71%	66.7%	37.8%
	SM interior	2 507 225	799	0.32	0.00%	0.00%	100.0%
Pernanbuco	Capital	1 599 513	10 360	6.48	3.51%	37.5%	15.4%
	LG interior	2 872 156	2 792	0.97	0.10%	59.8%	93.5%
	SM interior	4 736 881	1 034	0.22	0.00%	0.00%	100.0%
Alagoas	Capital	996 733	3 987	4.00	1.58%	47.5%	11.9%
	LG interior	227 640	261	1.15	0.00%	0.00%	100.0%
	SM interior	2 076 562	362	0.17	0.00%	0.00%	100.0%
Sergipe	Capital	614 577	3 180	5.17	0.83%	25.3%	27.5%
	LG interior	272 877	40	0.15	0.00%	0.00%	100.0%
	SM interior	1 308 208	157	0.12	0.00%	0.00%	100.0%
Bahia	Capital	2 883 682	11 582	4.02	3.98%	34.7%	16.4%
	LG interior	3 137 964	4 428	1.41	0.20%	81.4%	96.6%
	SM interior	9 022 491	2 641	0.29	0.00%	0.00%	100.0%
Minas Gerais	Capital	2 479 165	16 739	6.75	5.11%	35.6%	31.7%
	LG interior	6 608 202	16 518	2.50	7.23%	71.8%	54.6%
	SM interior	11 505 989	12 194	1.06	1.83%	89.4%	93.7%
Esp. Santo	Capital	348 268	4 146	11.90	2.56%	65.4%	19.6%
	LG interior	2 022 488	3 611	1.79	0.18%	78.8%	96.2%
	SM interior	1 468 610	1 034	0.70	0.00%	0.00%	100.0%
Rio De Jan.	Capital	6 429 923	40 378	6.28	7.42%	37.7%	39.2%
	LG interior	7 860 741	16 550	2.11	7.98%	82.6%	60.6%
	SM interior	2 078 515	3 035	1.46	2.90%	96.5%	83.8%
São Paulo	Capital	11 821 873	54 978	4.65	7.01%	35.5%	65.3%
	LG interior	20 588 317	47 241	2.29	13.25%	79.5%	76.5%
	SM interior	10 950 963	11 825	1.08	0.00%	0.00%	100.0%
Parana	Capital	1 848 946	10 738	5.81	3.68%	41.4%	26.7%
	LG interior	3 825 638	7 283	1.90	1.39%	65.4%	75.3%
	SM interior	5 322 881	4 093	0.77	0.00%	0.00%	100.0%
Santa Cat.	Capital	453 285	3 604	7.95	1.15%	62.3%	54.6%
	LG interior	2 504 181	6 380	2.55	1.30%	76.9%	82.7%
	SM interior	3 676 788	3 559	0.97	0.07%	86.3%	98.8%
RG Do Sul	Capital	1 467 816	13 068	8.90	3.40%	35.5%	36.0%
	LG interior	3 865 596	8 504	2.20	5.52%	78.2%	48.0%
	SM interior	5 830 631	5 730	0.98	0.00%	0.00%	100.0%
MG Do Sul	Capital	832 352	2 900	3.48	0.86%	40.5%	36.2%
	LG interior	424 478	967	2.28	0.13%	82.2%	89.1%
	SM interior	1 330 439	1 022	0.77	0.00%	0.00%	100.0%
Mato Grosso	Capital	569 830	2 279	4.00	0.79%	53.4%	40.8%
	LG interior	594 533	628	1.06	0.00%	0.00%	100.0%
	SM interior	2 017 750	1 579	0.78	0.00%	0.00%	100.0%
Goias	Capital	1 393 575	7 915	5.68	1.29%	31.4%	57.0%
	LG interior	1 892 869	1 615	0.85	0.01%	63.4%	98.6%
	SM interior	3 147 604	2 381	0.76	0.09%	100.0%	100.0%
Dist. Federal	Capital	2 789 761	8 299	2.97	1.14%	37.2%	63.3%

LM interior—interior large municipalities ≥ 100 000 inhabitants; SM interior—interior small municipalities < 100 000 inhabitants

^a^Physician per 1000 population

There is a high migratory flow of physician from capitals, LG interior and LM interior for the capitals of the Brazilian states, with special focus on the Southeast region or to the capital of the same region of the medical school graduation: 26.1% of the physician from capitals of the North region migrated to capital cities of the Southeast region; 36.1% of the physician from LM interior of the Southeast region migrated to some capitals of the same region. Other similar examples can be seen in Additional file 1: Table S1.

## Discussion

In December of 2017, Brazil had 447.321 [17] physicians and a population of 207,660,929 [18] inhabitants, which corresponded to a density of 2.17 physicians per 1000 inhabitants. The distribution of these professionals in the national territory was unequal [18], with the North (1.09 physicians per 1000 inhabitants) and Northeast (1.3) regions below the national ratio. In the seven Northern states, the ratio varied from 0.91 to 1.51. In the Northeast, the state of Maranhão had the lowest ratio of the country, with 0.79 physicians per 1000 inhabitants. The Southeast, with 2.75 physicians per 1000 inhabitants, the South, with 2.18, and the Central-West region, with 2.20, were above the national average.

In 2014, there were 241 active medical schools in Brazil, which together offered approximately 20,340 vacancies and were located mostly in the Southeast (99 schools, 41.0%) and Northeast (61 schools, 25.3%). Among the states, São Paulo (41 schools, 17%), Minas Gerais (34 schools, 14.1%), Rio de Janeiro (17 schools, 7.0%), Paraná (15 schools, 6.2%) and Rio Grande do Sul (15 schools, 6.2%) had the most medical schools. Currently, there are medical schools in all 27 federative units [17].

In the present study, 176 schools that trained physicians in Brazil between the years considered (1980 and 2014) were headquartered in 103 different municipalities. Of these, 25 were located in the state capitals and the Federal District, 63 were in large interior municipalities and 14 were in small interior municipalities.

Notably, among the 14,382 physicians who graduated from medical schools located in small interior municipalities (those with less than 100,000 inhabitants), the large majority (93.4%) did not stay at these locations. Most physicians who graduated from private medical schools also migrated (67.8%) regardless of the size of the municipality where the school was located.

Given the trend towards the interiorization and privatization of medical education in Brazil [19], the results of this study allow us to draw preliminary conclusions about the relationship between the location and nature of the school and the non-migration of physicians.

If adopted as an isolated measure, the opening of new, mostly private programmes in municipalities in the interior of the country in regions with a scarcity or a lower density of physicians per inhabitant may not directly affect the permanence of the graduated physicians in these locations. In this sense, the analysis has limitations because the study included a low number of physicians who graduated in small municipalities in the Northeast, North, Central-West and South regions because the internalization of medical schools is a recent phenomenon in Brazil.

The less-intense migration of women compared to men corroborates the literature, which indicates that the female gender is less prone to territorial relocation [20]. Thus, the feminization of medicine in Brazil [21] takes on increased relevance in the different aspects of the observed migration process, with a possible future positive effect on the non-migration of physicians.

The state capitals retained more physicians who graduated there. Although they had a smaller proportion than the capitals, the large cities of the country seemed to be more attractive than small cities to the physicians who graduated in these locations, which experienced a mass migration of physicians who graduated from their schools.

In Brazil, it was observed that the 39 cities with more than 500,000 inhabitants concentrated 30% of the population and 60% of the physicians, which directly affected the low physician/inhabitant ratio in the small municipalities. For example, in locations with 5000 to 10,000 inhabitants, the ratio is 0.28 physicians per 1000 residents. In municipalities with between 10,000 and 20,000 residents, the ratio is 0.36, whereas in municipalities with between 20,000 and 50,000 residents, the ratio is 0.64 physicians per 1000 inhabitants [15].

The results presented in Table 4 and in Additional file 1: Table S1 suggest that there is a high migratory flow between the capitals of the Brazilian states, especially with high influence of the state capitals from Southeast region; this region concentrates the largest number of medical residency programs in Brazil [14]. The few municipalities with less than 100,000 inhabitants, with the potential to train physicians, presented little retention capacity, losing their graduates to other regions. This situation has contributed to maintaining the low average per capita medical rate in these localities.

In this study, we also observed that there is a higher migration rate for physicians coming from private schools. This result may raise a warning about the new interiorization policies of medical schools in Brazil, a process that has resulted in an increase in the opening of private schools in the interiors of the states; one of the arguments used by the managers of this policy was that there could be retention of doctors in the host cities of medical schools and their surroundings.

In a systematic review realized by Willis–Shattuck et al. [22] to examine motivating factors that would reduce medical migration both within and across countries, the following factors were listed: financial incentives, career development, continuing education, hospital infrastructure, resource availability, hospital management and personal recognition or appreciation. A more recent systematic review identified similar variables with a high degree of similarity between selected studies; however, workload and autonomy seem to be specific to physicians’ choice of workplace between the public and private sectors [23].

A study that analysed geographical mobility of general practitioners in rural Australia found that in small towns of < 5000 residents had the highest risk of leaving rural practice. Mobility rates were significantly higher for GPs who had worked in a location for under 3 years and those working as either contracted or salaried employees, and somewhat higher for international medical graduates [24].

Mobility of US Rural Primary Care Physicians During 2000–2014 was analysed. The researchers noted that county-level physician mobility was higher for counties that lacked a hospital (absolute increase = 5.7%), had a smaller population size, and had lower primary care physician supply, but area level economic and demographic factors had little impact. Their conclusion was that outcomes were notably poorer in the most remote locations and those already having poorer physician supply and professional support [25].

This study may be considered a starting point for the required in-depth analysis of migratory patterns of physicians in Brazil. By using the database of administrative and professional records, it was possible to characterize the variables associated with the medical school and with the physicians who did or did not migrate.

Due to the sole focus on the comparison of the current address and graduation location, the present study did not analyse the migratory flow of physicians as a whole and did not consider the effect of the birthplace on relocation. Because this study was a cross-sectional study, it was not possible to capture possible intermediate moves and destinations over time or define whether the current location of the physician was definitive. In a classic study from 1978 on the flows and stocks of nurses and physicians, Mejía [26, 27] highlighted the difficulties of defining whether a professional’s migration was “permanent” or “temporary” based on secondary data.

Another limitation of the analysis is the concept of migration adopted in this study. The movement of a physician from one state to another may not represent a large displacement when it occurs between cities near the state border and can underestimate large displacements within the same state.

In this study, it was not possible to isolate the effect of the location of the medical school in relation to other cultural, employment, and socioeconomic factors that can influence migration.

Future studies on the subject may consider the characteristics of the place of origin (prior to obtaining the medical degree) and the current location of the professionals who migrated. For this purpose, more refined methods will be required, such as methods that use migration networks to establish possible migratory patterns.

## Conclusion

More than half of the physicians who graduated between 1980 and 2014 are living and/or working in federative units different from the unit where the medical school from which they graduated was located.

The considerable and dynamic internal migration of physicians in Brazil (defined in this study as the relocation or mobility of professionals from one state to another) seems to contribute to imbalances in the distribution, supply and availability of physicians in the country, whose motives are not yet fully clear.

When compared with physicians who remained in the state in which they graduated, there was a difference in the profiles of the physicians who migrated regarding individual factors, such as age, gender, time since graduation and specialty, which could partially explain the decision to migrate.

The probability of migration ratio was greater in small municipalities of the Southeast region and in the states of Tocantins, Acre and Santa Catarina, which also experienced a more pronounced migration of physicians.

To guide adequate human resource policies in health, new studies are required that consider the socioeconomic level of the location, the establishment of services and employment and continued training opportunities among other possible factors for the non-migration of physicians.

The “push” and “pull” mechanisms that motivate the internal migration of physicians are complex and multifactorial and can have consequences that require more research. For instance, qualitative studies should be performed to examine the migration networks and flows that are able to capture the controlled turnover (i.e., the supply of jobs to physicians from the outside) to meet the needs of health services and to offset retirement and other forms of replacement and exchange of professionals. More studies are also needed on voluntary turnover, which is when the physician leaves a certain place in search of better living conditions, wages or career prospects without a previous offer.

Similarly, the motivations for temporary migration should be explored by human resources policies, which can produce benefits by aggregating value to the professional and the local health system upon the physician’s return. This situation is in contrast to permanent migration, which can represent the transfer of human capital from one place to another and thus weaken the capacity of local health systems [2, 27–30].

Finally, research efforts should focus on monitoring the future movement of physicians in Brazil and other countries to identify factors related to internal migration and non-migration of these professionals and contribute to the conception, planning and assessment of public policies of human resources in health.

## Additional file

Additional file 1: **Table S1.** Origin and destination of the physicians who graduated in Brazil between 1980 and 2014. (DOCX 33 kb)
