# Supplementary material for: Internal migration of physicians who graduated in Brazil between 1980 and 2014
Source: Hum Resour Health. 2018 May 2;16:21. doi: 10.1186/s12960-018-0286-8 (PMC5930743; doi:10.1186/s12960-018-0286-8)
Supplement: Supplementary file 1 — Table S1. Origin and destination of the physicians who graduated in Brazil between 1980 and 2014. (DOCX 33 kb) [file 12960_2018_286_MOESM1_ESM.docx]

Additional file 1: Table S1. Origin and destination of the physicians who graduated in Brazil between 1980 and 2014.

|  |  | **DESTINATION** | | | | | | | | | | | | | | | | | | | | | | | | | | | | | | | | Total |
| --- | --- | --- | --- | --- | --- | --- | --- | --- | --- | --- | --- | --- | --- | --- | --- | --- | --- | --- | --- | --- | --- | --- | --- | --- | --- | --- | --- | --- | --- | --- | --- | --- | --- | --- |
| **ORIGIN** |  | **NORTH** | | | | | | **NORTHEAST** | | | | | | **SOUTHEAST** | | | | | | | **SOUTH** | | | | | | **CENTRAL-WEST** | | | | | | |  |
|  |  | **Capital** | | **LM interior** | | **SM interior** | | **Capital** | | **LM interior** | | **SM interior** | | **Capital** | | **LM interior** | | **SM interior** | | | **Capital** | | **LM interior** | | **SM interior** | | **Capital** | | **LM interior** | | **SM interior** | | |  |
|  | **M** | **N** | **%** | **N** | **%** | **N** | **%** | **N** | **%** | **N** | **%** | **N** | **%** | **N** | **%** | **N** | **%** | | **N** | **%** | **N** | **%** | **N** | **%** | **N** | **%** | **N** | **%** | **N** | **%** | | **N** | **%** |  |
|  | **NORTH** |  |  |  |  |  |  |  |  |  |  |  |  |  |  |  |  | |  |  |  |  |  |  |  |  |  |  |  |  | |  |  |  |
|  | **Capital** | 820 | 13.6 | 732 | 12.1 | 326 | 5.4 | 475 | 7.9 | 187 | 3.1 | 124 | 2.1 | 1576 | 26.1 | 539 | 8.9 | | 127 | 2.1 | 131 | 2.2 | 110 | 1.8 | 79 | 1.3 | 547 | 9.1 | 90 | 1.5 | | 164 | 6027 | 6027 |
|  | **LM interior** | 90 | 11.4 | 16 | 2 | 105 | 13.3 | 101 | 12.8 | 77 | 9.8 | 62 | 7.9 | 87 | 11 | 44 | 5.6 | | 18 | 2.3 | 4 | 0.5 | 5 | 0.6 | 2 | 0.3 | 120 | 15.2 | 18 | 2.3 | | 40 | 789 | 789 |
|  | **SM interior** | 64 | 8.2 | 19 | 2.4 | 66 | 8.5 | 44 | 5.7 | 34 | 4.4 | 47 | 6 | 106 | 13.6 | 61 | 7.9 | | 12 | 1.5 | 3 | 0.4 | 10 | 1.3 | 9 | 1.2 | 173 | 22.3 | 38 | 4.9 | | 91 | 777 | 777 |
|  | **NORTHEAST** |  |  |  |  |  |  |  |  |  |  |  |  |  |  |  |  | |  |  |  |  |  |  |  |  |  |  |  |  | |  |  | 0 |
|  | **Capital** | 345 | 2 | 100 | 0.6 | 123 | 0.7 | 2911 | 16.7 | 4961 | 28.4 | 3139 | 18 | 3099 | 17.7 | 977 | 5.6 | | 170 | 1 | 230 | 1.3 | 138 | 0.8 | 81 | 0.5 | 1015 | 5.8 | 58 | 0.3 | | 114 | 17461 | 17461 |
|  | **LM interior** | 32 | 1.2 | 4 | 0.1 | 5 | 0.2 | 1338 | 48.9 | 395 | 14.4 | 428 | 15.6 | 294 | 10.7 | 90 | 3.3 | | 22 | 0.8 | 15 | 0.5 | 13 | 0.5 | 4 | 0.1 | 89 | 3.3 | 2 | 0.1 | | 4 | 2735 | 2735 |
|  | **SM interior** | 0 | 0 | 0 | 0 | 0 | 0 | 0 | 0 | 0 | 0 | 0 | 0 | 0 | 0 | 0 | 0 | | 0 | 0 | 0 | 0 | 0 | 0 | 0 | 0 | 0 | 0 | 0 | 0 | | 0 | 0 | 0 |
|  | **SOUTHEAST** |  |  |  |  |  |  |  |  |  |  |  |  |  |  |  |  | |  |  |  |  |  |  |  |  |  |  |  |  | |  |  | 0 |
|  | **Capital** | 411 | 1.7 | 114 | 0.5 | 124 | 0.5 | 724 | 3 | 370 | 1.5 | 161 | 0.7 | 2438 | 10.1 | 12465 | 51.5 | | 4488 | 18.5 | 502 | 2.1 | 572 | 2.4 | 271 | 1.1 | 1149 | 4.7 | 170 | 0.7 | | 259 | 24218 | 24218 |
|  | **LM interior** | 565 | 0.9 | 202 | 0.3 | 258 | 0.4 | 1067 | 1.7 | 632 | 1 | 399 | 0.6 | 22342 | 36.1 | 19606 | 31.7 | | 9909 | 16 | 635 | 1 | 1145 | 1.8 | 813 | 1.3 | 2618 | 4.2 | 681 | 1.1 | | 1049 | 61921 | 61921 |
|  | **SM interior** | 114 | 0.9 | 38 | 0.3 | 60 | 0.5 | 272 | 2.2 | 170 | 1.4 | 130 | 1.1 | 3523 | 28.8 | 4004 | 32.7 | | 2200 | 18 | 180 | 1.5 | 254 | 2.1 | 209 | 1.7 | 703 | 5.7 | 136 | 1.1 | | 246 | 12239 | 12239 |
|  | **SOUTH** |  |  |  |  |  |  |  |  |  |  |  |  |  |  |  |  | |  |  |  |  |  |  |  |  |  |  |  |  | |  |  | 0 |
|  | **Capital** | 109 | 1.1 | 26 | 0.3 | 40 | 0.4 | 132 | 1.4 | 49 | 0.5 | 26 | 0.3 | 953 | 10 | 457 | 4.8 | | 114 | 1.2 | 503 | 5.3 | 4158 | 43.7 | 2522 | 26.5 | 223 | 2.3 | 79 | 0.8 | | 114 | 9505 | 9505 |
|  | **LM interior** | 142 | 0.8 | 39 | 0.2 | 58 | 0.3 | 158 | 0.9 | 60 | 0.3 | 45 | 0.3 | 1117 | 6.5 | 824 | 4.8 | | 224 | 1.3 | 4667 | 27.2 | 4467 | 26 | 4655 | 27.1 | 328 | 1.9 | 141 | 0.8 | | 239 | 17164 | 17164 |
|  | **SM interior** | 0 | 0 | 0 | 0 | 0 | 0 | 0 | 0 | 0 | 0 | 0 | 0 | 3 | 1.7 | 4 | 2.3 | | 1 | 0.6 | 23 | 13.1 | 53 | 30.1 | 89 | 50.6 | 0 | 0 | 1 | 0.6 | | 2 | 176 | 176 |
|  | **CENTRAL-WEST** |  |  |  |  |  |  |  |  |  |  |  |  |  |  |  |  | |  |  |  |  |  |  |  |  |  |  |  |  | |  |  | 0 |
|  | **Capital** | 163 | 3.7 | 56 | 1.3 | 93 | 2.1 | 172 | 3.9 | 46 | 1 | 51 | 1.2 | 840 | 19 | 524 | 11.9 | | 134 | 3 | 104 | 2.4 | 127 | 2.9 | 55 | 1.2 | 858 | 19.4 | 572 | 13 | | 618 | 4413 | 4413 |
|  | **LM interior** | 4 | 1.2 | 3 | 0.9 | 2 | 0.6 | 1 | 0.3 | 0 | 0 | 0 | 0 | 50 | 15.2 | 41 | 12.4 | | 19 | 5.8 | 7 | 2.1 | 15 | 4.5 | 14 | 4.2 | 128 | 38.8 | 9 | 2.7 | | 37 | 330 | 330 |
|  | **SM interior** | 1 | 0.4 | 0 | 0 | 0 | 0 | 2 | 0.8 | 0 | 0 | 0 | 0 | 9 | 3.7 | 1 | 0.4 | | 0 | 0 | 0 | 0 | 1 | 0.4 | 0 | 0 | 211 | 86.8 | 15 | 6.2 | | 3 | 243 | 243 |

**LM interior –** Interior large municipalities ≥ 100,000 inhabitants; **SM interior** – Interior small municipalities < 100,000 inhabitants.
